# A novel co-production of cadaverine and succinic acid based on a thermal switch system in recombinant Escherichia coli

**DOI:** 10.1186/s12934-022-01965-4

**Published:** 2022-11-24

**Authors:** Siyuan Gao, Jiachen Lu, Tongtao Wang, Sheng Xu, Xin Wang, Kequan Chen, Pingkai Ouyang

**Affiliations:** State Key Laboratory of Materials-Oriented Chemical Engineering, College of Biotechnology and Pharmaceutical Engineering, Nanjing Tech University, Nanjing, 211816 Jiangsu China

**Keywords:** Cadaverine, Succinic acid, Dynamic regulation, Two stage process, Metabolic engineering

## Abstract

**Background:**

Polyamide (nylon) is an important material, which has aroused plenty of attention from all aspects. PA 5.4 is one kind of nylon with excellent property, which consists of cadaverine and succinic acid. Due to the environmental pollution, bio-production of cadaverine and succinic acid has been more attractive due to the less pollution and environmental friendliness. Microbes, like *Escherichia coli*, has been employed as cell factory to produce cadaverine and succinic acid. However, the accumulation of cadaverine will cause severe damage on cells resulting in inhibition on cell growth and cadaverine production. Herein, a novel two stage co-production of succinic acid and cadaverine was designed based on an efficient thermos-regulated switch to avoid the inhibitory brought by cadaverine.

**Results:**

The fermentation process was divided into two phase, one for cell growth and lysine production and the other for cadaverine and succinic acid synthesis. The genes of *ldhA* and *ackA* were deleted to construct succinic acid pathway in cadaverine producer strain. Then, a thermal switch system based on pR/pL promoter and CI857 was established and optimized. The fermentation conditions were investigated that the optimal temperature for the first stage was determined as 33 ℃ and the optimal temperature for the second stage was 39 ℃. Additionally, the time to shifting temperature was identified as the fermentation anaphase. For further enhance cadaverine and succinic acid production, a scale-up fermentation in 5 L bioreactor was operated. As a result, the titer, yield and productivity of cadaverine was 55.58 g/L, 0.38 g/g glucose and 1.74 g/(L·h), respectively. 28.39 g/L of succinic acid was also obtained with yield of 0.19 g/g glucose.

**Conclusion:**

The succinic acid metabolic pathway was constructed into cadaverine producer strain to realize the co-production of succinic acid and cadaverine. This study provided a novel craft for industrial co-production of cadaverine and succinic acid.

## Introduction

Nylon polyamides (PA) polymerized by repeating diacid and diamine compounds, are widely used in multiple fields. 1,5-diaminopentane (cadaverine), an important material monomer, could be applied into synthesis of polyamide (PA), which is a major engineering material with various excellent advantages such as high toughness, strength and resistance to abrasion [1]. PA 5.X widely applied into various industries, including plastics, fibers, rubbers, foams and other fields, is synthesized by cadaverine and aliphatic diacids, whose market was assessed up to 220 million by 2022 [2]. However, fossil fuel was the main raw material for cadaverine production in traditional method, which led to plenty of environment problem during the production process. In contrast, synthesis of bio-based materials has become more attractive with the development of bio-manufacturing. As a result, the bio-production of monomer of nylon polyamides (cadaverine and succinic acid) has gradually become mainstream production method.

Recently, biosynthesis of cadaverine has been widely reported, which could be divided into two aspects. One method is bio-transformation where L-lysine was employed as substrate and converted into cadaverine in the presence of lysine decarboxylase and PLP. The other is fermentation pathway based on microbe cell factory. Because L-lysine is the precursor of cadaverine, the cadaverine synthetic pathway based on L-lysine synthetic pathway were the diaminopimelic acid (DAP) route in bacteria and plants and the α-aminoadipic acid pathway in most fungi and some archaea, respectively [3]. In procaryotic organism system, cadaverine is synthesized through the glycolysis pathway, tricarboxylic acid cycle, L-lysine synthesis pathway and DAP pathway with renewable carbon sources like glucose as substrate [4]. To enhance production of cadaverine, multiple metabolic engineering strategies were researched and employed into improve metabolic flux towards lysine, decrease synthesis of byproducts and enhance supply of cofactor [5, 6]. However, these metabolic modification often led to unbalanced cellular metabolic network, resulting in inhibition of cell growth and restriction of production [7]. In addition, the problem that the inhibitory effect of cadaverine will severely impact cell activity and limit cadaverine titer, is still unsolved [8]. Last but not least, the emission of carbon dioxide from decarboxylation of L-lysine to form cadaverine is an environmental issue that cannot be ignored.

To address the problems of cadaverine production mentioned above, a novel co-production of cadaverine and succinic acid strategy was designed based on dynamic regulation strategy. Dynamic regulation strategy is a novel metabolic engineering strategy, could which mainly applied into two aspects: pathway redirection and pathway balance [9]. Dependent on sensor/regulator system, the dynamic regulation strategy could effectively adjust cellular metabolism according to the changing environment. Therefore, the expression of target genes will be turned on only when they are needed in engineered strains [9, 10]. L-alanine is an important amino acid while it could severely affect cell growth and limit the ultima yield in the fermentation. Zhou et al. reported a thermo-regulated switch that dynamically regulated the expression of L-alanine dehydrogenase, which transformed pyruvate to form alanine [11]. Similar strategies were reported to dynamically regulate expression of target genes, such as *icd* encoding isocitrate dehydrogenase [12], *pfkA* encoding phosphofructokinase-1 [13] and *pyc* encoding pyruvate carboxylase [14]. Therefore, the problem of antagonism between cell growth and production could be solved by dynamic regulation strategy [15].

**Fig. 1:**
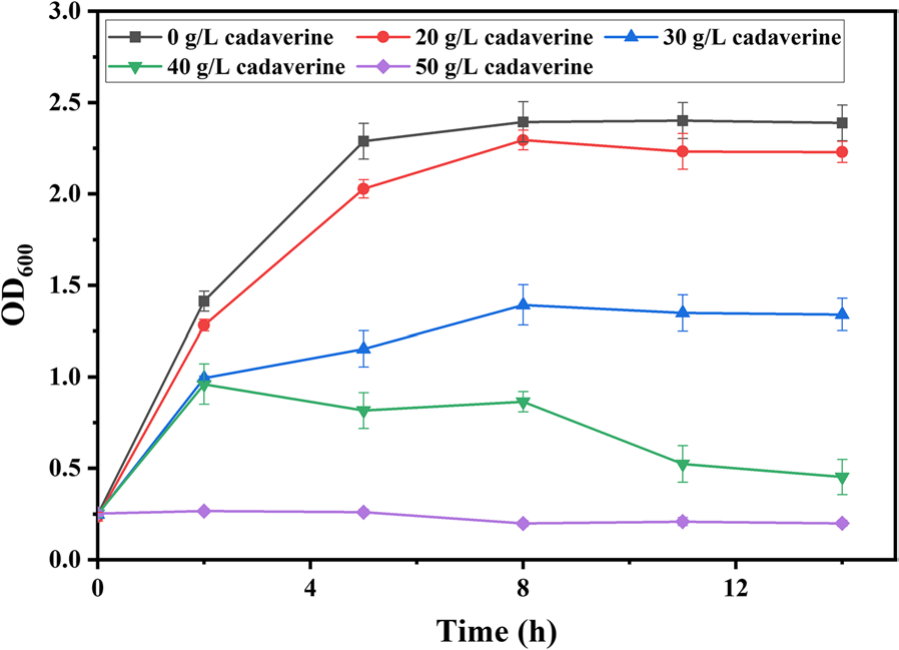
The inhibitory effect of cadaverine on cell growth. *E. coli* NT1003 was cultured in 0 g/L, 20 g/L, 30 g/L, 40 g/L and 50 g/L cadaverine for 14 h

In this study, the cadaverine producer strain was engineered to possess the ability to synthesize succinic acid. Then, a thermo-regulated switch was constructed to separate fermentation process into two stages, the one for lysine accumulation and the other for synthesis of cadaverine, which could effectively avoid the inhibitory effect caused by accumulation of cadaverine (Fig. 1). The effects of thermal switch on cadaverine production were investigated and the conditions for thermos-regulated cadaverine production was optimized. Finally, the novel strategy was operated in a 5 L of bioreactor for co-production of cadaverine and succinic acid.

## Materials and methods

### Strains and media

The plasmids used in this study were listed in Table 1. The strains used in this study are listed in Table 1. To construct succinic acid producer strain, the genes of *ldh* and *ackA* were knocked out from *E. coli* NT1003 by CRISPR-cas9 system. The procedures were described as Xin Wang et al. [6]. *E. coli* trans1 T1 used to construct recombinant plasmids was cultured in Luria–Bertani (LB) medium, consisting of 10 g/L peptone, 5 g/L yeast extract, 5 g/L NaCl. The seed medium for *E. coli* NT1003 and its recombinant strains included 10 g/L ammonium sulfate, 10 g/L yeast extract, 5 g/L peptone, 5 g/L sodium glutamate, 5 g/L sucrose, 0.3 g/L methionine, 0.3 g/L threonine, 77 mg/L MnSO_4_·H_2_0, 1 g/L MgSO_4_·7H_2_O with appropriate concentration of antibiotics. The fermentation medium consisted of 20 g/L Glucose, 10 g/L (NH_4_)_2_SO_4_, 5 g/L yeast extract, 5 g/L peptone, 0.5 g/L KCl, 1 g/L MgSO_4_·7H_2_O, 32 mg/L FeSO_4_·7H_2_O, 32 mg/L MnSO_4_·H_2_O, 86 mg/L ZnSO_4_·7H_2_O, 77 mg/L CuSO_4_, 60 mg/L Vitamin B1, 10 mg/L Nicotinamide, 30 μg/L Biotin, 0.3 g/L Threonine, 0.1 g/L Methionine. The antibiotic used in this study was kanamycin with the concentration of 50 mg/L. The fermentation medium of bioreactor contained 10 g/L ammonium sulfate, 10 g/L yeast extract, 5 g/L peptone, 0.5 g/L KCl, 1.6 g/L MgSO_4_·7H_2_O, 32 mg/L FeSO_4_·7H_2_O, 32 mg/L MnSO_4_·H_2_O, 86 mg/L ZnSO_4_·7H_2_O, 77 mg/L CuSO_4_·H_2_O, 0.3 g/L methionine, 0.3 g/L threonine, 60 mg/L vitamin B1, 30 μg/L biotin and 30 g/L initial glucose. The feeding medium included 600 g/L glucose and 400 g/L ammonium sulfate.

**Table 1:** List of *E. coli* strains and plasmids used in this study

Strains and plasmids	Genotype—descriptions	Reference
Strains		
* E. coli* Trans1-T1	F-φ80(lacZ)ΔlacX74hsdR (r_k_^−^, m_k_^−^) ΔrecA 1398endA 1 tonA	Transgen
* E. coli* NT1003	L-lysine hyper-producing strain	[6]
* E. coli* BL21-1-gfp	*E. coli* BL21(DE3), pRL1:*gfp*. Kan	This work
* E. coli* BL21-2-gfp	*E. coli* BL21(DE3), pRL2:*gfp*. Kan	This work
* E. coli* BL21-3-gfp	*E. coli* BL21(DE3), pRL3:*gfp*. Kan	This work
* E. coli* BL21-4-gfp	*E. coli* BL21(DE3), pRL4:*gfp*. Kan	This work
* E. coli* NT3201	*E. coli* NT1003Δ*ldhA*	This work
* E. coli* NT3202	*E. coli* NT1003Δ*ackA*	This work
* E. coli* NT3203	*E. coli* NT1003Δ*ldhA*Δ*ackA*	This work
* E. coli* KAC	*E. coli* NT3203, P_trc_:cadA. Kan	This work
* E. coli* KARL3	*E. coli* NT3203, P_RL3_:cadA. Kan	This work
* E. coli* KARL4	*E. coli* NT3203, P_RL4_:cadA. Kan	This work
Plasmids		
pET28a-gfp	Cloning *gfp* gene into *Bam*H I and *Hin*d III site of pET28a	
pRL1-gfp	One-step cloning pRL1 promoter to replace T7 promoter and lac operator in pET28a-gfp	This work
pRL2-gfp	One-step cloning pRL2 promoter to replace T7 promoter and lac operator in pET28a-gfp	This work
pRL3-gfp	One-step cloning pRL3 promoter to replace T7 promoter and lac operator in pET28a-gfp	This work
pRL4-gfp	One-step cloning pRL4 promoter to replace T7 promoter and lac operator in pET28a-gfp	This work
pRL3-cadA	One-step cloning *cadA* gene to replace *gfp* gene in pRL3-gfp	This work
pRL4-cadA	One-step cloning *cadA* gene to replace *gfp* gene in pRL4-gfp	This work
Ptrc-cadA	One-step cloning ptrc promoter to replace pRL3 promoter in pRL3-cadA	This work
sgRNA-*ΔldhA*	Vector for deletion of *ldhA* gene of the *E. coli*; Sm^r^	This work
sgRNA-*ΔackA*	Vector for deletion of *ackA* gene of the *E. coli*; Sm^r^	This work

### Construction of plasmids and characterization of thermal switch

The pRL1, pRL2, pRL3 and pRL4 were four promoters containing different part of λ phage pRL promoter [15]. The four promoters were amplified by PCR from pPL451 and then cloned in plasmid pET28a-*gfp* to form recombinant pRL1-*gfp*, pRL2-*gfp*, 2 pRL3-*gfp* and pRL4-*gfp*. Then the four recombinant plasmids were transformed into *E. coli* BL21 to characterize the effect of thermal switch on *gfp* expression. After culturing for 12 h on a LB medium plate with 100 mg/L of kanamycin, the transformants were selected and pre-cultured in 5 mL of LB medium for 12 h. The pre-cultured strains were added into a 500 mL-flask containing 100 mL of LB medium with 1% (v/v) inoculum size. Then, four strains were cultured under 30 °C, 37 °C and 42 °C, respectively. After cultured for 12 h, the four strains were collected by centrifugation and re-suspended into 5 mL of PBs 7.0 buffer. The biomass and was measured by OD_600_. The strength of four promoters was measured by fluorescence intensity at an excitation wavelength of 485 nm and emission wavelength of 526 nm. The expression level of *gfp* was evaluated by the value of a.u. (485 nm/526 nm) divided by OD_600_.

### Optimization of thermal switch system

Due to the existence of thermal switch, the fermentation was divided into two isolated parts. One part was used for cell growth and lysine accumulation and the other for cadaverine synthesis. The temperatures for cell growth and for cadaverine synthesis were investigated. In cell growth phase, four temperatures of 30 ℃, 33 ℃, 35 ℃ and 37 ℃, were selected. The recombinant strains and parent strain were cultured for 8 h of cell growth phase. Then, the temperature was shifted to 42 °C for cadaverine production for 16 h. In cadaverine production, four temperatures of 35 ℃, 37 ℃, 39 ℃ and 42 ℃, were selected to find the optimum temperature for cadaverine production. All strains were pre-cultured in 33 °C for 8 h and then cultured in the indicated temperature for cadaverine production. In addition, the time to switch temperature was also studied. Four time points, 8 h, 16 h, 19 h and 22 h, were decided to study. All experiments were conducted in 500 mL shake flask. The biomass and the concentration of glucose, lysine and cadaverine were monitored during the fermentation.

### Two-stage fermentation in 5 L fermenter

The cadaverine production under thermos-regulated switch was operated in a 5 L-bioreactor (BLBIO-5GJ-2, Bailun, Shanghai, China). The engineered strain was cultured in 100 mL of LB medium for pre-culture for 8 h and transferred into 500 mL of seed medium for 12 h. Then, all seed culture was transferred into 5 L reactor. The pH remained at 6.9 and the culture temperature was kept at 33 °C for 48 h. Then, the temperature was shifted to 40 °C for expression of lysine decarboxylase to synthesize cadaverine. During the fermentation, the glucose was continuously fed to maintain under 10 g/L.

### Analytical methods

The biomass was monitored by measuring OD_600_. The concentration of lysine and glucose was measure by SBA biosensor [6]. The cadaverine and succinate acid was analyzed by HPLC [6, 16].

## Results and discussion

### Design of a two-stage production of cadaverine and succinic acid based on a thermal switch system

**Fig. 2:**
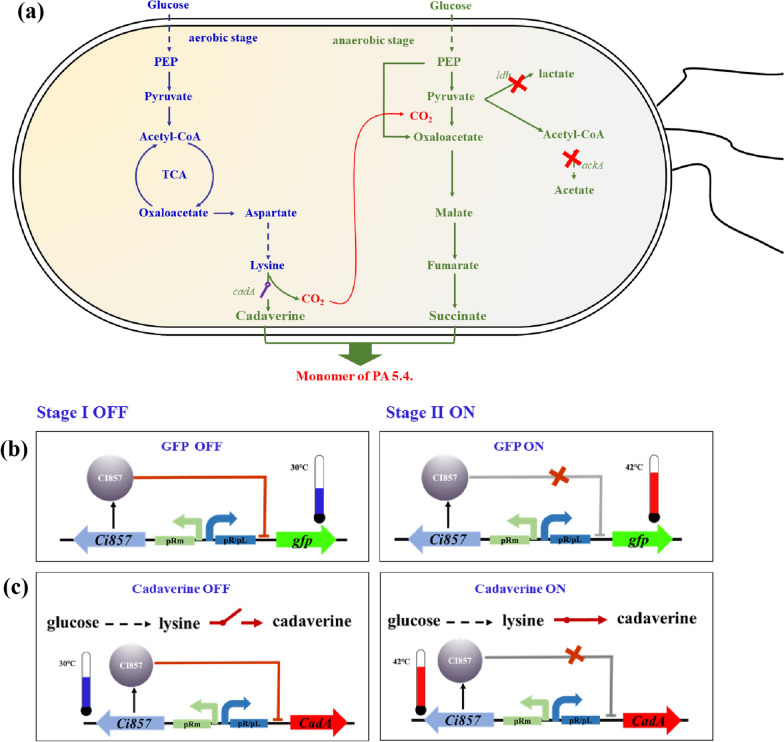
An overview of the novel craft of cadaverine and succinic acid based on a thermos-regulated switch. **a** Schematic representation of the metabolic networks of cadaverine and succinic acid production. Dynamic control of **b** *gfp* and **c** *cadA* expression by a thermo-regulated switch

PA 5.4 consisted of cadaverine and succinic acid, both of which have been produced by microbial factory [17, 18]. However, the inhibitory effect caused by high level of intracellular cadaverine has been reported to significantly limit cadaverine production [8]. According to Fig. 1, 30 g/L of cadaverine had a severe impact on cell growth, leading to 50% loss of biomass. To solve the problem of product inhibition, a two-stage production strategy was designed based on dynamic regulation that the initial stage is for lysine accumulation and the latter is for cadaverine synthesis and succinic acid production (Fig. 2). For the two stage fermentation, the previous phase was designed as low temperature and aerobic stage, where the engineered strain grew and produced lysine while the latter stage was high temperature and anaerobic stage that this stage notably was under sealed status. Interestingly, the sealed status provides the anaerobic condition for succinic acid production and prevents spillage of carbon dioxide from decarboxylation of lysine that the carbon dioxide will be ingeniously employed that it will be in situ fixed and utilized as carbon source and be dissolved into fermentation broth to form carbonic acid to neutralize the alkalinity from synthesis of cadaverine. By this way, we could achieve that reducing the use of acids in fermentation, co-producing the monomers of PA 5.4. at the same time and utilizing carbon dioxide from decarboxylation of lysine to enhance atom economy of the whole process.

### Construction of succinic acid metabolism pathway in cadaverine producer strain

**Fig. 3:**
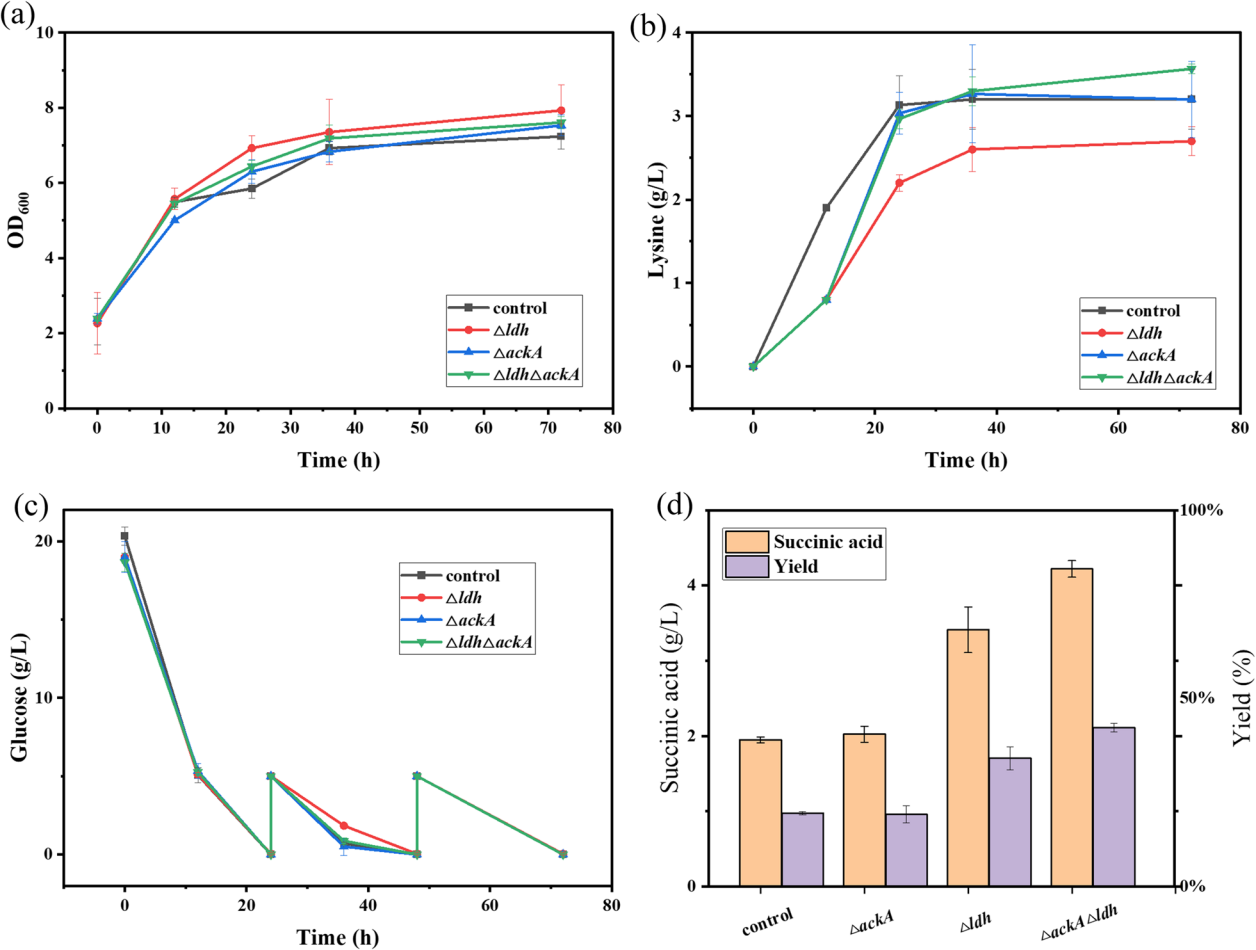
Construction of succinic acid producer strain based on NT1003. **a** Cell growth curve, **b** lysine production, **c** consumption of glucose and **d** succinic acid production of engineered and parent strains

To realize co-production of succinic acid and cadaverine, metabolism pathway of succinic acid was constructed in the cadaverine producer strain, NT1003. According to Jung et al. deletion of *ldhA* and *ackA* could avoid accumulation of lactate and acetate [19]. Therefore, the D-lactate dehydrogenase coded by *ldhA* and acetate kinase coded by *ackA* was deleted from genome of *E. coli* NT1003 by CRISPR (Fig. 2a). The cell growth, production of lysine and succinic acid of engineered strains were characterized. As is shown in Fig. 3, the curve of cell growth and lysine production of NT3201, NT3202 and NT3203 resembled that of the control, which illustrated that deletion of *ldhA* and *ackA* have little influence on cell growth and lysine production. After 72 h fermentation, the titer of succinic acid of NT1003 was mere 1.25 g/L while the titers of NT3201, NT3202 and NT3203 were 2.02 g/L, 3.41 g/L and 4.22 g/L, respectively, which were 1.62, 2.73 and 3.38 times of that of the control. The results reflected that deletion of *ldhA* and *ackA* could effectively improve production of succinic acid. Thus, the engineered strain NT3203 was employed as chassis cell for further study.

### Construction and characterization of the thermal switch system

**Fig. 4:**
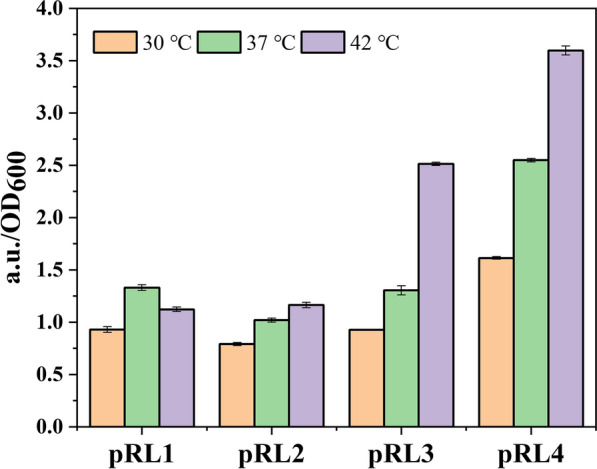
Construction and screening of the thermo-regulated switch. The thermos-regulated system was identified by *gfp*

The thermal switch system consisted of the repressor CI857 and promoter pR/L from λ bacteriophage [12]. The mechanism was described in Fig. 2b that when environmental temperature was under low status (30 °C), the repressor CI857 will bind to promoter pR/L, resulting in transcription cease. Once temperature reached a high bound (42 °C), the repressor CI857 will lose its activity and will be detached from the promoter-repressor complex, and the gene regulated by pR/L could be normally expressed. However, the pR/L sequence was not clearly reported. To investigate the effects of pR/L regions from genome of λ phage, four different lengths of pR/L were constructed and cloned in pET28a-*gfp*, in which *gfp* gene was employed to characterized the strength of promoters. Three temperatures, 30 °C, 37 °C and 42 °C, were selected to characterize the thermal switch in *E. coli.* As shown in Fig. 2, pRL2, pRL3 and pRL4 exhibited that expression intensity of *gfp* increased with increasing temperature. Additionally, expression intensity of pRL2 was lower than that of pRL3 and pRL4. The value of a.u./OD_600_ of pRL2 was only about 50% and 33% of that of pRL3 and pRL4, respectively. Thus, the promoter pRL3 and pRL4 were selected for further study (Fig. 4).

**Fig. 5:**
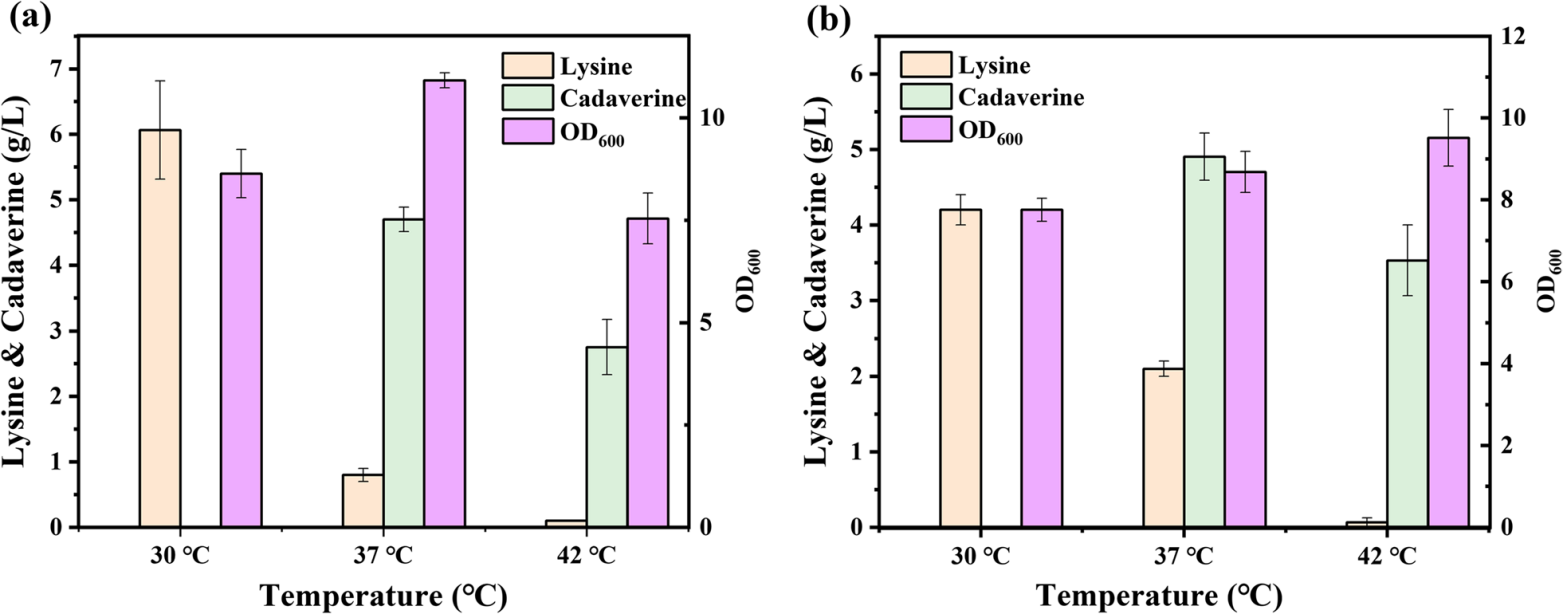
Application of thermo-regulated switch in cadaverine production. The lysine decarboxylase was regulated by (**a**) pRL-3 and (**b**) pRL-4

To applying the thermal switch in cadaverine production, the *gfp* gene was replaced by *cadA* encoding lysine decarboxylase which converts L-lysine to form cadaverine. The recombinant plasmids pRL3-cadA and pRL4-cadA were transformed into engineered *E. coli* NT3203 to form thermo-regulated *E. coli* NT3203/pRL3-cadA (KARL3) and *E. coli* NT3203/pRL4-cadA (KARL4). As is showed in Fig. 5, both KARL3 and KARL4 showed thermos-regulated lysine/cadaverine production. Under low temperature (30 ℃), *cadA* was not expressed and the main product was lysine. When temperature increased to 37 ℃, the *cadA* was gradually expressed and KARL3 and KARL4 began to produce cadaverine. Cadaverine was the main product in KARL3 and KARLC4 and little lysine could be detected under 42 °C. In addition, the biomass of KARL3 was affected by temperature that the value of OD_600_ under 37 °C was higher than that of 30 °C and 42 °C. However, this phenomenon was not observed in KARL4. Taking everything into consideration, the thermo-regulated cadaverine production was established and realized in KARL4, which was determined for cadaverine production optimization.

### Optimization of thermal switch system in cadaverine production

In order to compare routine cadaverine production with dynamic regulated cadaverine production, the strain KAC, a cadaverine producer where *cadA* was constitutively expressed, was used as the control. Due to application of thermal switch, the whole fermentation process was divided into two phase, low temperature phase and high temperature phase. According to above study, low temperature phase, in which the cell was in growing and producing lysine, also was called cell growth phase. After cell growth phase, low temperature will be changed to high temperature for cadaverine production and this phased was identified as cadaverine production phase.

**Fig. 6:**
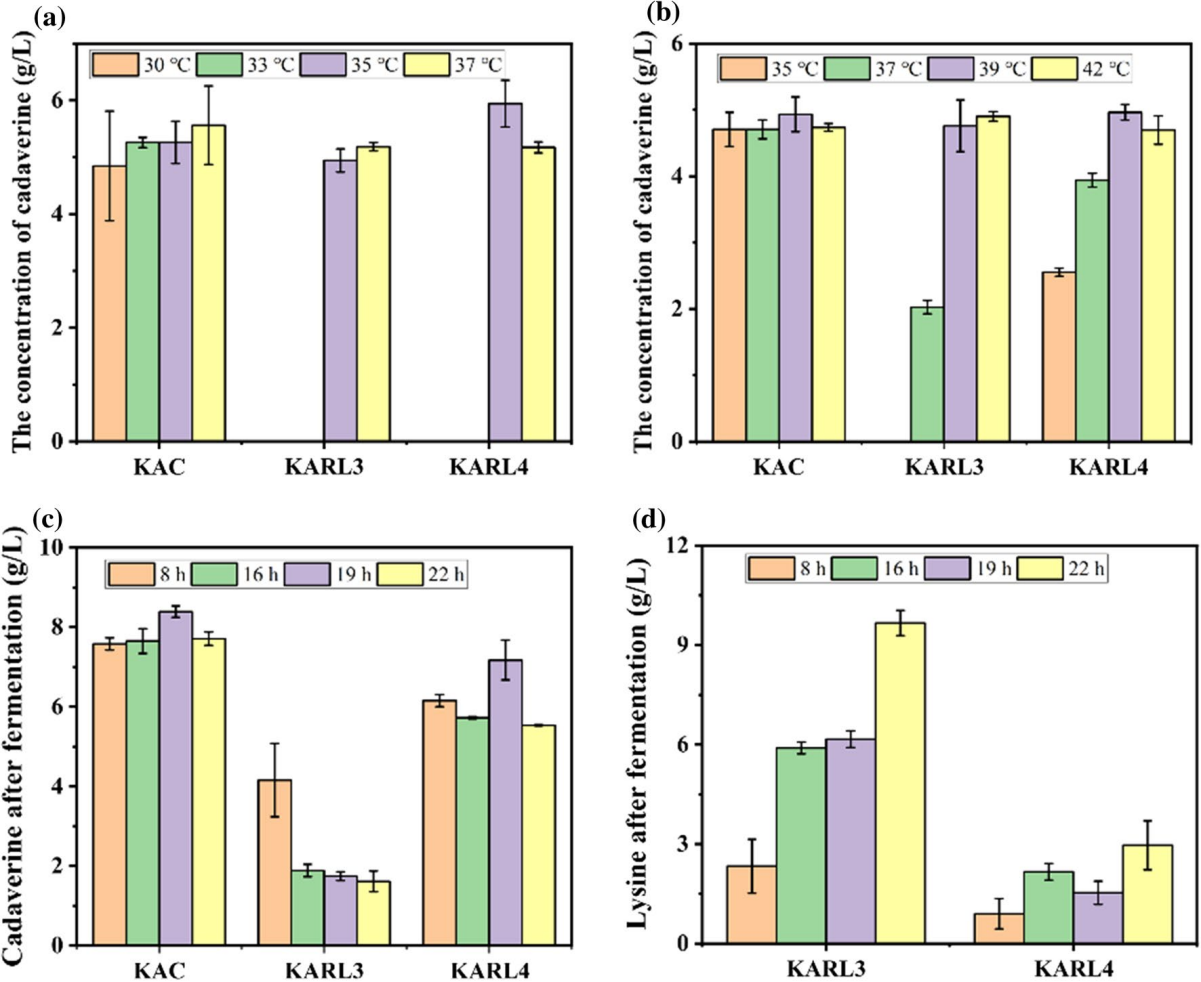
Optimization of thermo-regulation switch. **a** Temperature optimization for cells growth; **b** Temperature optimization for cadaverine production; **c**, **d** Time optimization for shifting temperature. The concentration of **c** cadaverine and **d** lysine after fermentation

In first stage, four different temperatures, 30 ℃, 33 ℃, 35 ℃ and 37 ℃, were selected to investigate. The titers of cadaverine at four temperatures are similar whatever in KAC or in KARL3 or in KARL4 (Fig. 6a). However, biomass of recombinant strains was found preference under 37 °C, which was reported the optimal temperature for *E. coli* growth (Table 2). Furthermore, we found that the *cadA* gene would not be expressed at 33 °C. Thus, 33 °C was determined as temperature for cell growth.

**Table 2:** Comparison of fermentation data obtained with different temperatures for the first stage

Temperature	30 ℃		33 ℃		35 ℃		37 ℃	
OD_600_&Lys	OD_600_	Lys (g/L)	OD_600_	Lys (g/L)	OD_600_	Lys (g/L)	OD_600_	Lys (g/L)
KAC	6.81 ± 0.57	0	7.99 ± 0.71	0	5.84 ± 0.48	0	7.76 ± 1.10	0
KARL3	7.16 ± 1.22	2.51 ± 0.45	7.95 ± 1.17	3.26 ± 0.37	6.96 ± 2.89	0.50 ± 0.3	8.28 ± 0.19	0.30 ± 0.17
KARL4	5.92 ± 0.74	2.10 ± 0.26	6.87 ± 0.77	2.20 ± 0.55	7.53 ± 0.47	0.13 ± 0.05	7.95 ± 0.45	0

After cell growth for 8 h, the temperature for cadaverine production was investigated. As shown in Fig. 6b and Table 3, effects of different temperatures on cadaverine production seems to not obvious in KAC. The most salient distinction between KARL3 and KARL4 is the existence of cadaverine in system at 35 °C that 2.55 g/L cadaverine was detected in KARL3 while no cadaverine could be detected in KARL4, which illustrated that leakage expression of *cadA* occurred at 35 °C in KARL3. The contradiction of cadaverine at 35 °C in KARL3 could be attributed to the temperature switch. The thermal switch system relied on the different activity of repressor CI857 under different temperature. Although leakage expression existed at 35 °C, repressor CI857 cannot be completely inactivated when the temperature was raised to 35 °C. Additionally, the titers of cadaverine in KARL3 and KARL4 at 39 °C and 42 °C were similar that means the repressor was complete inactivated at 39 °C. Therefore, we chose 39 °C for cadaverine production.

**Table 3:** Comparison of fermentation data obtained with different temperatures for the second stage

Temperature	35 ℃			37 ℃			39 ℃				42 ℃		
OD_600_&Lys	OD_600_	Lys (g/L)		OD_600_	Lys (g/L)		OD_600_	Lys (g/L)		OD_600_		Lys (g/L)	
Time	20 h	8 h	20 h	20 h	8 h	20 h	20 h	8 h	20 h	20 h		8 h	20 h
KAC	4.49 ± 0.43	0	0	4.19 ± 0.38	0	0	4.36 ± 0.14	0	0	5.35 ± 0.08		0	0
KARL3	4.22 ± 0.45	0.83 ± 0.11	3.17 ± 0.29	4.84 ± 0.36	0.83 ± 0.05	2.73 ± 0.11	4.39 ± 0.10	0.93 ± 0.11	0.75 ± 0.40	4.82 ± 0.42		0.90 ± 0.01	0.90 ± 0.01
KARL4	5.00 ± 0.18	0.83 ± 0.05	2.14 ± 0.10	4.76 ± 0.34	0.80 ± 0.01	1.60 ± 0.57	4.37 ± 0.65	0.77 ± 0.11	0.20 ± 0.26	5.51 ± 0.44		0.80 ± 0.10	0.80 ± 0.1

The time to switch temperature is an important factor of thermos-regulated cadaverine production. The cadaverine fermentation was divided into two isolated parts, one for cell growth accompanying with synthesis of lysine and the other for cadaverine production. When cell status was switched from cell growth to cadaverine production, the cell growth would be limited, which was the common contradiction between cell growth and cell production in bio-manufacturing [15, 20]. Thus, it was necessary to study the effects of time to switch temperature on cadaverine production. Four time points, including 8 h, 16 h, 19 h and 22 h after inoculation, were selected for further research. In Fig. 6c, d, the switch time point affected cadaverine production. When the time to switch temperature was at 8 h, the cadaverine titer was the highest compared with that at 16 h, 19, and 22 h in KARL3 (Fig. 6c), and the residual lysine showed reverse tends (Fig. 6d). However, the relationship between the time changing temperature and the titer of cadaverine was not found in KARL4. Out of expectation, the cadaverine titers in KARL3 and KARL4 showed a clear gap (Fig. 6c, d), that the cadaverine titer of KARL4 was higher than that of KARL3 under all conditions. In fact, the strength of pRL4 was proved to be higher than pRL3, which meant that more lysine decarboxylase could be synthesized in same time, resulting in more cadaverine in fermentation. In KARL4, the difference of cadaverine titer in four groups was not obvious, while the most lysine remained when time to change temperature was 22 h, which illustrate that there was not enough time for synthesis of lysine decarboxylase and transformation lysine to cadaverine. Thus, a fed-batch fermentation was conducted in KARL4 with glucose as substrate.

The fed-batch fermentation in shake flask lasted 32 h, where the glucose, lysine and cadaverine were monitored. In early 24 h, The concentration of lysine in KARL4 gradually increased and was up to 8.93 g/L as time goes on. After shifting temperature to 39 °C, the lysine decreased to 4.63 g/L and 3.82 g/L of cadaverine was obtained at 32 h in KARL4, while the cadaverine was the only product in KAC and its titer reached 7.04 g/L at 32 h. Although the cadaverine titer in KAC was higher than that in KARL4, there was 4.63 g/L of lysine in fermentation system, which was not transformed into cadaverine. Meanwhile, the time point to switch temperature was 24 h and there were 8 h for expression of *cadA* to produce lysine decarboxylase and synthesize cadaverine. In a word, the time set aside was insufficient, which was an important issue to consider in fermentor production. However, the activity of KAC or KARL4 decreased gradually, such as consumption rate of glucose, due to a variety of reasons. Hence, the thermos-regulated cadaverine production was operated in a 5 L-bioreactor.

### Two-stage fermentation for cadaverine and succinic acid coproduction in a 5 L bioreactor

**Fig. 7:**
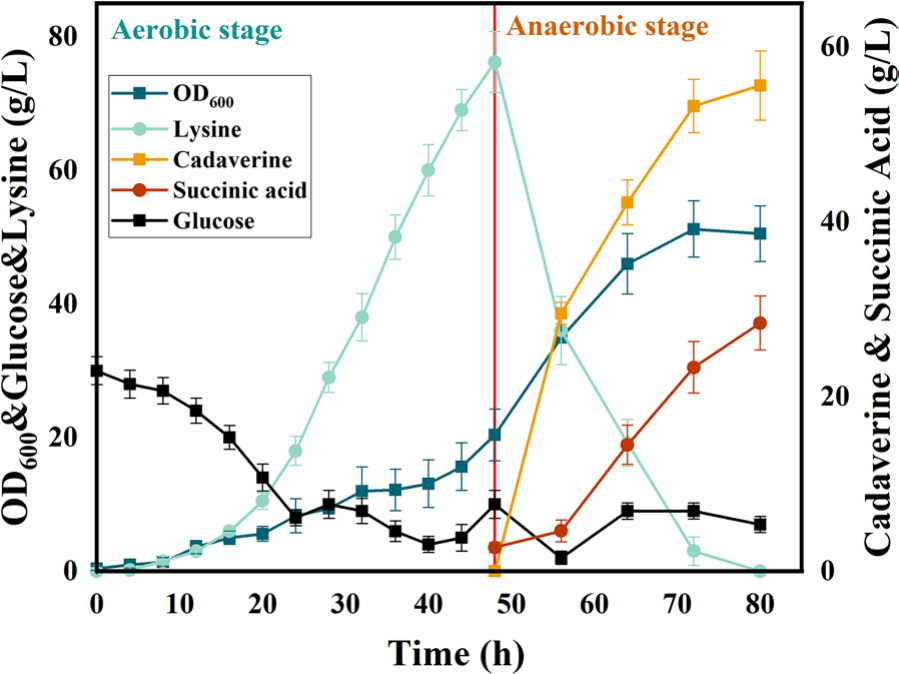
Cadaverine production by a thermos-regulated switch in a 5 L bioreactor. The red line represented the time when the culture was shifted from 33 to 39 °C

In order to efficiently produce cadaverine, a scale-up fermentation was conducted in a 5 L bioreactor. The fermentation was divided into two stage, cell growth stage and cadaverine production stage, which is same as shake flask fermentation. As is showed in Fig. 7, the concentration of lysine increased up to 76.2 g/L in first stage (0–48 h). The OD_600_ of engineering strain was mere 20.4 at 48 h for the low temperature about 33 °C. The temperature was shifted to 39 °C from 33 °C at 48 h. The biomass of KARL4 increased rapidly up to 51.2 at 72 h, which was 2.5 times of that at 48 h. In cadaverine production stage from 48 to 80 h, the concentration of lysine quickly decreased and cadaverine was synthesized. The titer of cadaverine was up to 55.58 g/L at 80 h and the productivity of cadaverine was 1.74 g/(L·h) in the second stage (The time for cadaverine produce was determined as 32 h). The overall cadaverine yield from glucose was 0.38 g/g in whole process.

In addition, the fermentation system was shifted to anaerobic fermentation when the temperature was raised from 33 to 39 °C. Although the lysine synthesis would be limited by the anaerobic condition, succinic acid was accumulated in this system, which could neutralize the alkalinity of cadaverine resulting in reducing the addition of hydrochloric acid. Furthermore, succinic acid is another raw material of PA5.4 besides cadaverine. The series production of cadaverine and succinic acid was realized through a thermos-regulated switch. The titer of succinic acid was showed in Fig. 7 that 28.39 g/L of succinic acid was detected in the final 32 h. The productivity and overall yield of succinic acid was 0.89 g/(L·h) and 0.19 g/g glucose in the fermentation, respectively.

In fact, there are much effort to complete. According to Jiang et al. the titer of succinic acid has been reported up to 101.2 g/L by two stage fermentation [21]. However, the strain KARL4 was engineered based on lysine producer, which was inadequacy ability for succinic acid production, while the culture strategy was not suitable for succinic acid fermentation as well. The dynamic regulation could whether be applied into the strain engineering to enhance production of succinic acid. For example, down-regulated the expression of competing enzymes in succinic acid synthesis pathway, including *ldhA*, *adhE*, *ackA* and *ptsG* [22, 23] while the key genes like *pyc *[24], *mdh *[25] could be overexpressed to enhance synthesis of succinic acid through dynamic regulation strategy. However, the results in our study presented a promising production for succinic acid and cadaverine. The similar research was operated by wang et al. that the lysine decarboxylase was expressed in succinic acid producer and the lysine was added into system to form cadaverine for regulation of pH. According to wang et al., the titers of cadaverine and succinic acid were 22.0 g/L and 21.2 g/L, which was only 39.58% and 74.67% of that in our study.

Additionally, the cadaverine/succinic acid stage was greatly complex and particular, in which the lysine was transformed into cadaverine with release of carbon dioxide. Meanwhile, the fermentation system was sealed, leading to gradual increase of the carbon dioxide concentration. The utilization of carbon dioxide to synthesize succinic acid has been reported [26, 27]. In previous research, ^13^C-tracer analysis was conducted to verify that carbon dioxide could be utilized by KARL4 [1].

## Conclusion

This study described a novel two-stage production strategy based on thermos-regulated switch, to realized coproduction of cadaverine and succinic acid. An engineered strain was constructed for succinic acid production with a cadaverine producer as chassis cell. Then, dynamic regulation of lysine decarboxylase was employed to avoid accumulation of cadaverine and effectively relieve inhibition caused by cadaverine as a result. The fermentation process was separated into two phase by thermos-regulated switch. The former stage was used for cell growth and lysine production, and the latter stage was responsible for synthesis of cadaverine and succinic acid. The titer, yield and productivity of cadaverine in a 5 L bioreactor was 55.58 g/L, 0.38 g/g glucose and 1.74 g/(L·h), respectively. In the whole fermentation, 28.39 g/L of succinic acid was obtained with yield of 0.19 g/g glucose. In this whole process, the acid use and emission of dioxide carbon could be effectively reduced and the atom economy could be improved as a result.

## Data Availability

All data generated or analyzed during this study are included in this article.
